# Microscale pressure measurements based on an immiscible fluid/fluid interface

**DOI:** 10.1038/s41598-019-56573-x

**Published:** 2019-12-27

**Authors:** Jing Yang, Xing Duan, Andrew K. Fraser, Mohammad Ikbal Choudhury, Andrew J. Ewald, Rong Li, Sean X. Sun

**Affiliations:** 1 Department of Mechanical Engineering, The Johns Hopkins University, Baltimore, MD 21218 USA; 2 School of Physical Science and Technology, Lanzhou University, Lanzhou, Gansu 730000 China; 3 Center for Cell Dynamics, Department of Cell Biology, The Johns Hopkins University School of Medicine, Baltimore, MD 21205 USA; 4 Institute of NanoBioTechnology (INBT), The Johns Hopkins University, Baltimore, MD 21218 USA

**Keywords:** Biophysical methods, Techniques and instrumentation

## Abstract

A method of microscale pressure measurement based on immiscible fluid/fluid interface is proposed. This method utilizes observed curvature changes in a fluid/fluid interface, and can accurately report hydraulic pressure in fluids at length scales of 10 microns. The method is especially suited for measuring fluid pressure in micro-scale biological samples. Using this method, we probe fluid pressure build up in epithelial domes, murine mammary gland organoids embedded in hydrogel, and lumen pressure in the developing mouse embryo. Results reveal that the pressure developed across epithelial barriers is on the order of 100~300 Pa, and is modulated by ion channel activity.

## Introduction

In biology, fluid pressure is a state variable that plays important roles in the mechanics of cells and tissues. In cells, the cytoplasmic hydraulic pressure is related to the osmotic pressure, and is carefully regulated by the cells^1^. In tissues, hydraulic pressure determines the flow behavior of the fluid phase in the extracellular matrix, and may impact processes such as cell movement and cell migration^2–4^. Indeed, cells are sensitive to hydraulic pressure^5,6^ and may adjust their gene expression and functional behavior accordingly. However, due to difficulties in measuring pressure, especially at small scales, the role of pressure in many biological processes remain unclear. During past decades, researchers have made efforts to develop different kinds of sensors and methods to measure pressure within microscale domains. Broadly, these pressure measurement methods can be divided into two categories, i.e., non-invasive or invasive. Non-invasive methods estimate pressure by applying external perturbation to generate mechanical deformations; a model is then used to obtain pressure. For instance, micropipette aspiration^7^ and atomic force microscopy (AFM)^8,9^ measure forces necessary to generate mechanical deformations, and pressure is obtained by modeling the deformation. A related method is to measure deformation in an elastic gel^10,11^ caused by an expanding cell or embryo. These methods often require additional modeling of fluid flow interacting with mechanical structures. Therefore, the accuracy of non-invasive methods depends on the mechanical models used to estimate pressure. In comparison, invasive methods are based on introducing a pipette directly into the domain of interest, and then convert pressure into measurement signals. For example, the barometer method^12–14^ and the servo-null method^15–17^ directly report pressure based on previous calibration. Currently, for cell-scale pressure measurements, the most often used invasive method is the servo-null method. The principle of servo-null method is to apply external pressure into a pipette filled with salt solution that has high electrical resistance, and the pipette is inserted into the domain of interest. It is assumed that when the applied external pressure is equal to the internal pressure of the domain, electrical resistance of the pipette equals to that when the pipette is free of any pressure. The accuracy of null-servo method may be affected by some factors, such as the ionic composition of the media, electrical resistance of the environment and properties of ionic diffusion.

**Figure 1:**
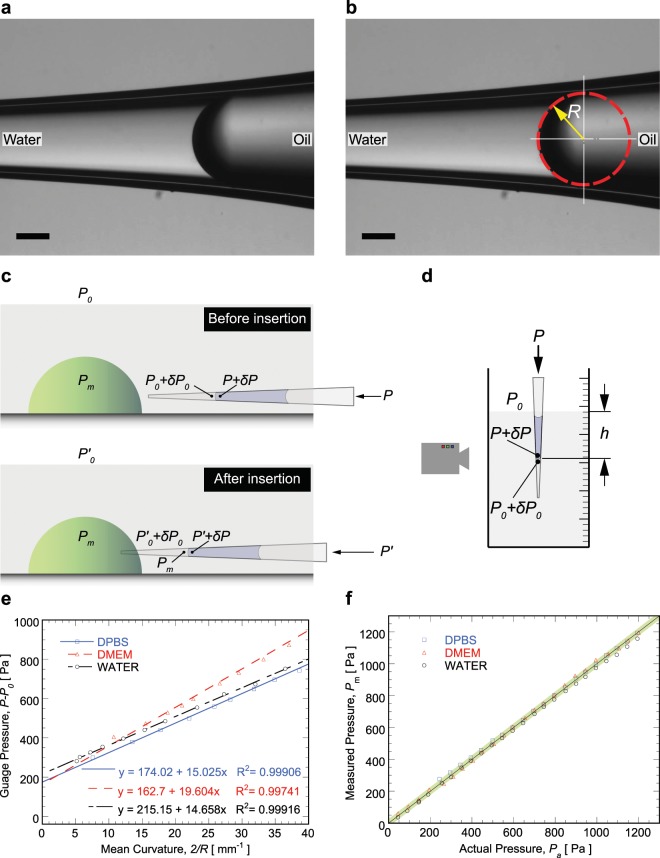
(**a**) Typical mineral oil/water interface. (**b**) Mean curvature of the interface is estimated through image processing, in which interface is regressed as part of a circle with radius *R*, and mean curvature is determined as 2/*R*. Scale bars are 100 *μ*m; (**c**) Schematic of pressure distribution before and after inserting pipette into the region interested. (**d**) Schematic of pressure distribution for validating the proposed method. (**e**) Calibration curves for determining surface tensions between mineral oil and different media (DPBS, DMEM, and pure water). (**f**) Validation results demonstrated with different media. Error band flanking diagonal line is 20 Pa.

Here, we developed a pressure measurement method based on curvature changes in an immiscible fluid/fluid interface (see Fig. 1(a)), which utilizes the Young-Laplace principle, The proposed method is simple, straightforward and does not require knowledge of the interfacial surface tension. The method is invasive, i.e., requires insertion of a pipette into the medium of interest. It also requires imaging, but a typical bench top microscope is sufficient. The method is quantitatively accurate up to 10 Pa. We apply this method to pressure measurements in epithelial domes, lumen pressure in artificial organoids, and a developing mouse embryo. Results show that chemical signals and ion channel function can modulate lumen pressure, suggesting that cells can actively regulate hydraulic pressure in tissues.

## Principle

The key component of the pressure sensor is a glass micropipette with a sharp tip, loaded with an immiscible fluid to the fluid in the domain of interest (Fig. 1(a)). So far, silicon oil, decane, and mineral oil have been used as the immiscible fluid, and mineral oil is the best due to its stability and ease of handling. The interface between immiscible fluids is then employed as a pressure indicator in terms of mean curvature, since the pressure difference Δ*P* between two sides of the interface follows Young-Laplace equation, i.e.,

1 $$\Delta P=\frac{2\sigma }{R}$$

in which, *σ* is surface tension between immiscible fluids, and 2/*R* is mean curvature of the interface (Fig. 1(b)). The tip of the pipette is inserted into the domain of interest, and the other end of the pipette is connected with a pressure chamber, in which the pressure can be adjusted with a syringe pump and monitored with a pressure transducer. Once surface tension, curvature of interface, and pressure on one side of interface is known, then pressure on the other side of interface can be obtained from Eq. (1).

Each pressure measurement involves two steps (Fig. 1(c)). In the first step (before pipette was inserted into the region of interest), assuming the atmosphere pressure is *P*_0_, and pressure on the front side of the interface (i.e., left side of the interface in Fig. 1(c)) is *P*_0_ + *δP*_0_, in which *δP*_0_ is the pressure difference (from the atmosphere) in the medium immediately outside of the region of interest. The pressure in the pressure chamber is *P*, and pressure on the back side of the interface (i.e. right side of the interface in Fig. 1(c)) is *P* + *δP*, where the pressure difference from pressure chamber to the right side of the interface is *δP*. Then, the pressure difference across the interface can be written as:

2 $$P+\delta P-({P}_{0}+\delta {P}_{0})=\frac{2\sigma }{{r}_{0}}$$

in which, *r*_0_ is the interface radius of curvature before insertion into the region of interest.

During the second stage (after the pipette is inserted into the region of interest), the pressure on the front side of interface is *P*_*m*_, i.e., the pressure to be measured. At the same time, the pressure in the pressure chamber may change to *P*′, and correspondingly the pressure on the right side of the interface is *P*′ + *δP* (in which, *δP* is mostly affected by the height of oil column in the pipette as well as the capillary pressure from the oil and air interface. Since before and after the pipette insertion, these factors are generally constant, therefore the change in *δP* can be neglected, and *δP* can be considered constant). Usually the position of interface in the pipette has negligible variation due to the small pressure being measured. If visible change of interface position occurs, the interface can be moved back to its original place by adjusting the pressure in the pressure chamber with syringe pump. If the atmospheric pressure at this moment changes to ${P}_{0}^{^{\prime} }$, then pressure adjacent to the region interested is ${P}_{0}^{^{\prime} }+\delta {P}_{0}$. The pressure difference *δP*_0_ is determined by the depth of the medium where the pipette is located. To insert the pipette into the region of interest properly, the pipette has to be in the focus during the operation, so before and after the pipette insertion its depth variation in the medium will not exceed a few microns, and it is acceptable to consider *δP*_0_ as a constant. So, the pressures in the pipette after insertion satisfy the equation:

3 $$P^{\prime} +\delta P-{P}_{m}=\frac{2\sigma }{{r}_{m}}$$

where, *r*_*m*_ is the interface radius of curvature after insertion into the region of interest.

After subtracting Eqs. (2) and (3) and rearranging, we obtain:

4 $${P}_{m}-({P}_{0}^{^{\prime} }+\delta {P}_{0})=2\sigma (\frac{1}{{r}_{0}}-\frac{1}{{r}_{m}})+(P^{\prime} -{P}_{0}^{^{\prime} })-(P-{P}_{0})$$

The left side of Eq. (4) is the relative pressure to be measured for the region of interest, and *P* − *P*_0_ and $P^{\prime} -{P}_{0}^{^{\prime} }$ on the right side are the gauge pressures detected by the pressure transducer in the pressure chamber before and after insertion, respectively. The “ghost term” ${P}_{0}^{^{\prime} }$ on both sides of Eq. (4) can cancel each other, but here they are left in for convenience.

Our pressure measurement method is validated by dipping the pipette tip into a fluid medium of known depth and pressure (see Fig. 1(d)). For validation, the procedure for measuring pressure is slightly different. First, the pressure sensor is dipped into a validation medium at a fixed depth, then the syringe-pump is used to change the immiscible interface between oil and validation medium to different positions. The interface image is recorded for each position, as well as the gauge pressure in the pressure chamber. Since pressures on the two sides of the interface satisfy Eq. (2), we can re-arrange to obtain

5 $$P-{P}_{0}=\delta {P}_{0}-\delta P+\frac{2\sigma }{r}$$

Left side of Eq. (5) is the gauge pressure in the pressure chamber, and 2/*r* is mean curvature of the interface. Using linear regression of gauge pressures versus mean curvature, surface tension *σ* (slope of the regression line), as well as *δP*_0_ − *δP* (intercept), can be obtained. Since *δP*_0_ is known according to the depth of the pipette tip into the validation medium, then *δP* can be estimated from the intercept *δP*_0_ − *δP*. After this, the pipette can be moved to different desired depth *h*, and *δP*_0_ (the pressure to be measured) is

$$\delta {P}_{0}=P-{P}_{0}+\delta P-\frac{2\sigma }{{r}_{m}}$$

in which, *P* − *P*_0_ is the gauge pressure of the chamber, and *δP* and *σ* have been obtained from the regression (Fig. 1(e)) in the previous step. The measured pressure *δP*_0_ is obtained from the mean curvature of the interface, and the measured pressure is compared with the corresponding known hydrostatic pressure *ρgh*, where, *ρ* is density of the validation medium, and *g* is gravity acceleration. After the pipette is dipped into the dish, the pipette tip is filled with the cell culture medium in the dish, which should be osmotically compatible with the cell. Additionally, each measurement takes less than half minute, so for such short time the diffusion of cell culture medium from the pipette into cell will not affect the measurement significantly. To rule out media composition effects, three validation media are presented in Fig. 1(e), i.e., DPBS (Dulbecco phosphate-buffered saline), DMEM (Dulbecco’s Modified Eagle Medium) and pure distilled water. The data shows that the surface tensions between mineral oil and DPBS, DMEM and pure water are 15.02 dynes/cm, 19.60 dynes/cm, and 14.66 dynes/cm, respectively. Fig. 1(f) shows that the measured pressures are very close to the actual known pressure. The absolute error (accuracy) is within ±10 Pa (highlighted error band) in a wide pressure range from 50 Pa to 1200 Pa in the experiment.

## Results and Discussion

### MDCK domes

Madin-Darby Canine Kidney (MDCK) cells are epithelial cells used extensively in studies of cell polarity, cell-cell adhesion, and collective cell motility, etc. When grown to confluence on impermeable substrates, MDCK cells can form fluid-filled domes. Here, we measure the pressure inside MDCK domes using our method under different conditions.

**Figure 2:**
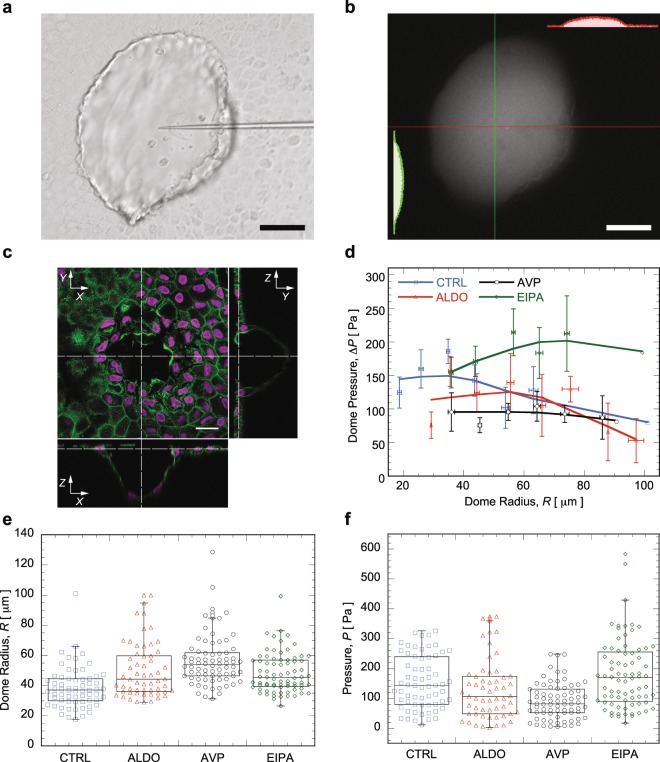
(**a**) Bright-field image of a MDCK dome inserted with a pipette (scale bar is 50 *μ*m). (**b**) MDCK dome injected with fluorescence dye for leaking verification, insets are fluorescence intensity distribution along highlighted lines. (**c**) Confocal fluorescence image of MDCK dome for illustrating its 3D structure, F-actins are labeled as green, and nuclei are labeled as magenta (scale bar is 20 *μ*m). (**d**) Pressure inside MDCK domes measured under the influence of different drugs as a function of dome size in terms of radius. (**e**) Dome size in terms of radius and (**f**) pressure inside MDCK domes under different drug treatment.

During the pressure measurement, the pipette tip is inserted into MDCK domes (see Fig. 2(a)) using a micromanipulator, and the size of the pipette tip is about 3 *μ*m in diameter, and the dome is about 20~100 *μ*m. After insertion, cells can self-seal the epithelium, and there is no significant fluid leak observed in the experiment. To verify the epithelium is sealed after pipette insertion, fluorescent dye (FITC dextran, 10 mM) was injected into the dome and monitored over time. Results (Fig. 2(b)) showed that 5 minutes after dye injection, the fluorescence remain inside the dome without leaking out. Fig. 2(c) shows the stained structure of fixed cells in the MDCK dome (green is F-actin, and magenta is nucleus), indicating a single layer of cells with typical epithelial morphology.

Fig. 2(d) plots the measured dome pressure as a function of dome radius, and also under treatment of several drugs, i.e., AVP (arginine vasopressin), ALDO (Aldosterone), and EIPA (Ethylisopropylamiloride). Dome radius is estimated from the image of the dome. The results indicate that dome pressure is around 100~300 Pa, which is consistent with values obtained by other studies^11,18,19^. It shows that pressure increases with dome radius for small size domes, and after a certain size, the pressure starts to decrease with increasing dome radius. This trend is consistent for domes treated with different drugs. Domes treated with ALDO or AVP have slightly lower pressure than untreated cells, while EIPA increases the dome pressure. Currently there is a lack of understanding of how dome pressure is modulated by cells and drugs. Fig. 2(e) also shows that domes treated with ALDO or AVP are statistically larger in size and have lower dome pressure, while there is no similar trend for the domes treated with EIPA (see Fig. 2(f)). The size of the dome is determined by fluid flux transported across the epithelium during dome growth. The current method does not measure fluid transport into the dome. Factors that cause domes to collapse are also unknown, and therefore it is not clear what determines the overall dome size. Causative reasons for pressure variations inside MDCK domes treated with different drugs need more investigation and is beyond the scope of this paper.

### Mammary gland organoids

A murine mammary gland organoid is a piece of mammary duct that contains epithelial and myoepithelial cells together with basement membrane components. The organoid can be cultured in 3D gels, and used as a model platform for studying the mechanisms that regulate normal growth and differentiation, as well as malignant transformation of mammary gland^20^. Three-dimensional embedded culture of mammary gland organoid is also an important tool for investigating branching morphogenesis and tumorigenesis, such as invasion and dissemination. However, few papers have studied the role of pressure during these aforementioned processes. Here, we present some experimental data about the pressure measurement in organoids with the proposed method.

**Figure 3:**
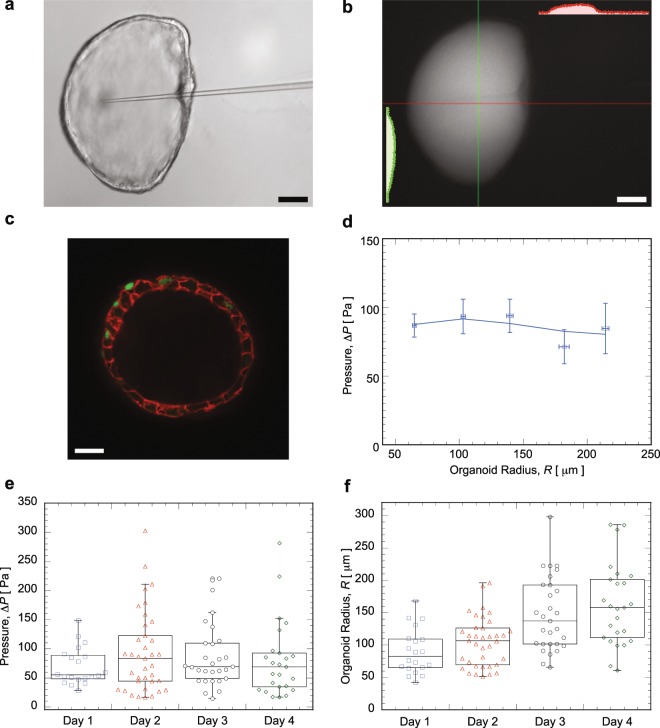
(**a**) Mammary organoid with a pipette inserted (scale bar is 50 *μ*m). (**b**) Fluorescence intensity distribution five minutes after the injection of dye without removing the pipette. (**c**) Confocal fluorescence image of an organoid, membrane is coded with red, while green is nucleus (scale bar is 20 *μ*m). (**d**) Pressure inside mammary gland organoids as a functions of organoid size in terms of radius. (**e**) Pressure inside mammary gland organoids at different days after lumen initiation. (**f**) Corresponding organoid size as a function of days after lumen initiation.

In this experiment, the pipette has to penetrate through the gel before insertion into organoids, and Fig. 3(a) shows a pipette tip inserted into an organoid embedded in gel. The tip of the pipette is around 3 *μ*m to ensure enough sensitivity, while still small enough to avoid disturbing the organoid. Mammary gland organoids presented here are treated with FGF2 and hydrocortisone. Similar to the case of MDCK domes, the dye assay was used to check for epithelial tightness (Fig. 3(b)). A confocal fluorescence image of organoid in Fig. 3(c) indicates that the lumen inside the organoid is surrounded by a layer of mammary gland cells tightly connected (cell membranes are in red, and nuclei are in green) to each other. Pressure as a function of organoid radius is plotted in Fig. 3(d), indicating that pressure changes little with size, and pressure is around 100 Pa on average. Organoid radius is estimated by fitting the outline of organoid as a circle according to its image recorded. Pressure inside the organoid has no significant variation with respect to the number of days after the initiation of lumen formation, and can be considered as constant over time (Fig. 3(e)), while the size of organoid grows with time (Fig. 3(f)).

### Mouse embryo

**Figure 4:**
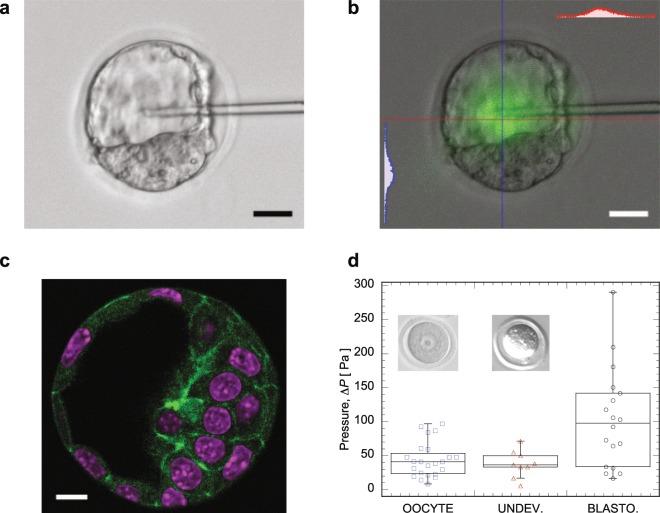
(**a**) Blastocyst with a pipette inserted (scale bar is 20 *μ*m) into the lumen. (**b**) Leaking verification experiment for blastocyst. Insets are fluorescence intensity profile along the highlighted lines (red and blue) across blastocyst. (**c**) Confocal fluoresence images of blastocyst, in which F-actin is stained as green, and DNA is stained as magenta (scale bar is 10 *μ*m). (**d**) Pressure measured for mouse oocyte at different stages, i.e., unfertilized oocyte, oocyte that is fertilized but not developed for some reasons, and blastocyst.

During embryonic development, hydraulic pressure might play important roles in determining embryo morphology, trigger activation of biochemical pathways, signal development^21^, etc. In the later stage of the mouse embryo, the fertilized oocyte develops into a blastocyst, which has a fluid-filled cavity surrounded by the inner cell mass inside the zona pellucida (Fig. 4(a,c)). The pressure measurement method proposed here is applied to measure the pressure in the blastocyst cavity. For consistency, fluorescent dye leak verification was conducted on blastocyst, and results are shown in Fig. 4(b). The procedure is the same as that for MDCK domes and mammary organoids, fluorescence dye was injected into blastocyst cavity with a pipette, then the pipette was kept inserting for more than 5 minutes, and there is no obvious dye loss observed in the experiment.

Fig. 4(d) presents the pressure measured in mouse embryos at different stages, i.e., unfertilized oocyte, fertilized-but-undeveloped oocyte, and blastocyst. According to the experimental data, the pressure inside the blastocyst is around 100 Pa. This is significantly higher than that for the unfertilized oocyte and the fertilized-but-undeveloped oocyte. These differences suggests that pressure is actively controlled by the cell, and might play an important role during the development of embryo.

## Conclusions

In this paper, we proposed a simple and robust method of pressure measurement based on curvature of an immiscible interface. The method does not require electrical measurements, and therefore does not depend on the ionic composition of the media. Furthermore, knowledge of interface surface tension is not necessary. Therefore, the results are robust and independent of system details. The method can also measure pressure in small volumes, potentially as small as a single cell cytoplasm. To achieve single cell resolution, pipettes would need to be significantly smaller and therefore other effects may come into play. For instance, when the pipette tip is smaller than 1 *μ*m and the measurement volume is small when compared to the pipette volume, pressure equilibration may become an issue.

The pressure measurement result shows that extracellular lumen pressure is tens to a few hundred pascals higher than background pressure (usually atmospheric pressure). This pressure difference is small, but on the per cell basis, it translates to 10–100 nN of force. This pressure difference is also balanced by tensions in the epithelia. Therefore cells likely are generating active tension in order to balance this pressure difference^22,23^. Pharmacological perturbations show that the lumen pressure is actively regulated by the cell. Our method provides another way to quantify these tensions in living epithelia or embryos, and can be useful for understanding the role of pressure in growth and development.

## Materials and Methods

All animals used in this research were handled in accordance with guidelines defined and approved by the Institutional Animal Care and Use Committee of Johns Hopkins University School of Medicine under protocol number M018M85.

### Experimental setup

**Figure 5:**
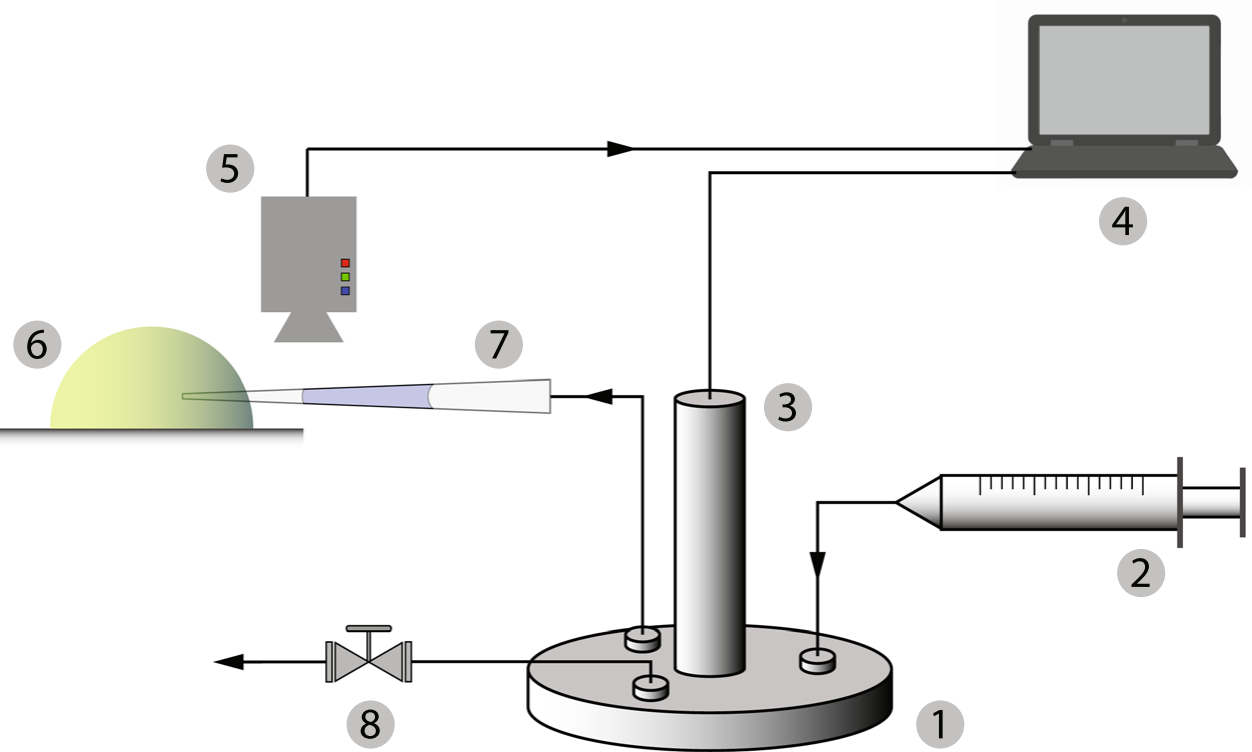
Experimental setup for pressure measurement. 1. Pressure chamber; 2. Air pump; 3. Pressure transducer; 4. Computer for collecting data; 5. Camera; 6. Object in interest; 7. Pipette pressure probe; 8. Pressure relief valve.

**Figure 6:**
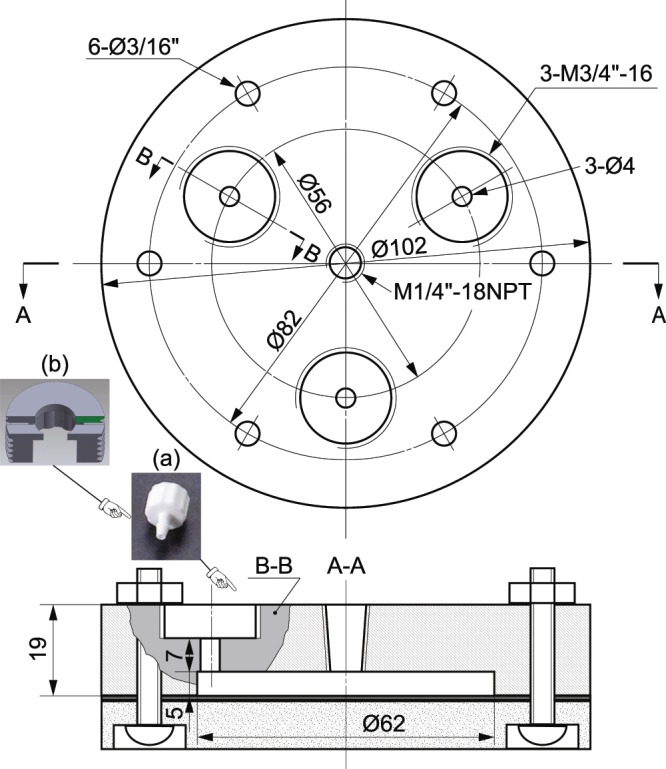
Stucture and dimensions of pressure chamber (units are millimeter, unless indicated). The assemble of bottom plate of chamber is indicated in the cross section A-A of the top part of chamber. The (**a**) Luer-to-tube connector is locked on the chamber by a (**b**) specially-designed screw manufactured by a 3D printer.

A typical experiment setup is shown in Fig. 5. A pressure chamber (specific structure and dimensions are shown in Fig. 6) is connected to a syringe pump (NE-1000, New Era Pump Systems, Inc., 10 ml syringe, B-D Company) that increases or decreases the pressure inside the pressure chamber. A pressure transducer (PX409, Omega Engineering, Inc.) measures the gauge pressure inside the pressure chamber. The pipette pressure probe back-connected with the pressure chamber is installed on a micromanipulator (MMO-202ND, Narishige International USA, Inc.) attached to a microscope (Axiovert 200, Carl Zeiss, Inc.). The pipette probe is pre-loaded with mineral oil. The oil/medium interface in the probe is recorded with a camera (GRAS 20S4M, Point Grey, Inc.) for later image analysis to estimate the curvature of the interface.

### Pressure chamber

The specific structure and dimensions of pressure chamber are presented in Fig. 6, and the pressure chamber is made of aluminum to reduce the weight, as well as ease of machining. After the assembly of pressure chamber, all the junctions were sealed with RTV sealant (DOWSIL 734, DOW, Inc.). Sealed pressure chamber was merged into a water tank and pumped to certain pressure, then checked visually if there is air bubble leaking, and monitor the pressure without significant drop.

### Micropipette preparation

**Figure 7:**
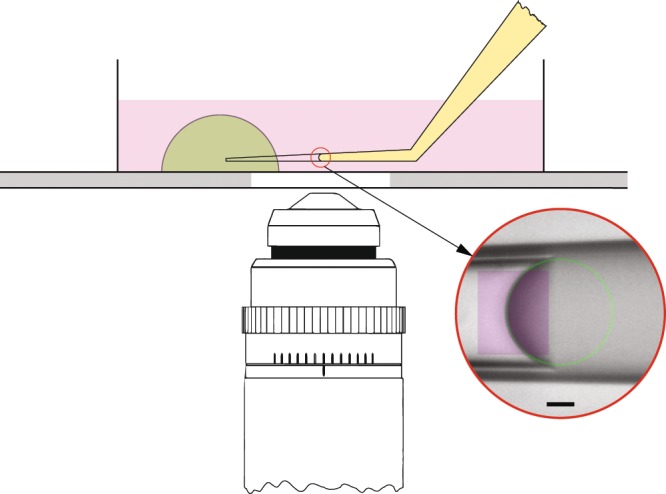
Schematic of pressure measurement to indicate that the bent region of micro-pipette is approximately parallel to the bottom of dish. Inset within red circle indicates a typical result of image processing of an immiscible fluid/fluid interface for estimating the curvature of the interface, in which, purple rectangle is the selected region of interest for the regression, and green circle is the regression result based on the portion of interface in the selected region. Scale bar is 10 *μ*m.

The micropipette for pressure measurement was pulled through platinum heating filament of pipette puller (P-97, Sutter Instrument Co., USA) from borosilicate glass tube (B100-75-15, Sutter Instrument Co., USA) according to certain parameter settings. A 20 to 35 degree bend in the taper of the pipette was created on the flame of a Bunsen burner, so the bent pipette tip is parallel to dish bottom (see Fig. 7) after being installed on the micro-manipulator with an incident angle. The pipette tip is generally parallel to the dish bottom. In addition, most of the interface is approximately spherical with a constant mean curvature, so small deviation from parallel will not affect the measurement significantly. The pipette tip was cut using a microforge (MF-830, Narishige International USA, Inc.) to make an opening of around 3 *μ*m at the tip. Mineral oil (M5904, Sigma-Aldrich, Inc.) of about 1 cm in length was loaded from the rear to the tip region of the pipette, and extra oil can be removed by a paper roll of Kimwipe. Then, the prepared micropipette was inserted into a micropipette holder, installed on the micro-manipulator, and ready for pressure measurement.

### MDCK domes

MDCK cells were seeded in a dish having glass bottom (Fluorodish, World Precision Instruments, INC.). The dish has 2 ml growth medium, i.e., Dulbecco’s modified Eagle’s medium (Corning DMEM with L-Glutamine, 4.5 g/L Glucose and Sodium Pyruvate) supplemented with 20% Fetal Bovine Serum (American Type Culture Collection, VA USA) and 1% penicillin strepomycin (Gibco, ThermoFisher Scientific). Cells were incubated at 37 °C in 5% CO_2_, and maintained by changing growth medium every two days; Once a confluent monolayer was formed, and domes were established, the dish was ready for pressure measurement. Before the pressure measurement, growth medium was replaced with DMEM medium supplemented with 1% Sodium Pyruvate and 25 mM hydroxyethyl-piperazine ethane sulphonic acid (HEPES), as well as the treating chemicals (such as, 0.01 *μ*M ALDO, 0.01 *μ*M AVP, and 1 *μ*M EIPA), and the dish was incubated for two hours.

### Murine mammary gland organoid

Mammary organoids were prepared according the published protocols^20,24^. Briefly, mammary glands were harvested from FVB mice 8–12 weeks of age. After enzymatic digestion of stromal tissue, epithelial duct fragments of 200–400 cell were isolated by differential centrifugation. These fragments, referred to as organoids, were embedded in a 3D Matrigel matrix. Organoids were cultured either in organoid media (DMEM-F12 with 1% insulin-transferrin-selenium, 1% penicillin-streptomycin, and 2.5 nM FGF2) or in media supplemented with hydrocortisone (1.1 *μ*M). The organoids were maintained at 37 °C and 5% CO_2_ for up to seven days.

### Mouse embryo

Female CD1 mice (8–10 weeks) were injected introperitoneally with 5.0 international units (IU) pregnant mare serum gonadotrophin (PMSG, Sigma-Aldrich, Inc.) to super-ovulate. Forty eight hours after PMSG injection, the mice received an injection of 5.0 IU human chorionic gonadotrophin (HCG, Sigma-Aldrich, Inc.). The mice start ovulation around 14 hours after HCG injection, metaphase II-stage (MII) oocytes were collected in the ampulla region of the oviduct and stored in human tubal fluid (HTF, Sigma-Aldrich, Inc.). Spermatozoa were retrieved from the epididymides of male adult CD1 mice and pre-incubated at 37 °C with 5% CO_2_ for one hour in HTF, which is covered with mineral oil. Spermatozoa were mixed with oocytes in the HTF media for *in vitro* fertilization, then fertilized oocytes were washed to remove the excess sperms and cultured at 37 °C with 5% CO_2_ in KSOM media (Sigma-Aldrich, Inc.), which is covered by paraffin oil.

### Image processing

The images of immiscible fluid/fluid interface recorded in the experiments were analyzed through image processing, and the code for image processing was designed in Mathematica language. The region of interest in the original image was cropped out, and then *EdgeDetect* algorithm was applied to obtain the outline of the interface. Some unwanted noise can be removed by applying *DeleteSmallComponents* algorithm and sorting *MorphologicalComponents*. The resultant outline of interface was regressed as a circle to estimate its curvature. A typical result is shown in the inset of Fig. 7.
